# A breast cancer-specific combinational QSAR model development using machine learning and deep learning approaches

**DOI:** 10.3389/fbinf.2023.1328262

**Published:** 2024-01-15

**Authors:** Anush Karampuri, Shyam Perugu

**Affiliations:** Department of Biotechnology, National Institute of Technology, Warangal, India

**Keywords:** breast cancer, QSAR (quantitative structure-activity relationship), GDSC^2^, machine learning, regression

## Abstract

Breast cancer is the most prevalent and heterogeneous form of cancer affecting women worldwide. Various therapeutic strategies are in practice based on the extent of disease spread, such as surgery, chemotherapy, radiotherapy, and immunotherapy. Combinational therapy is another strategy that has proven to be effective in controlling cancer progression. Administration of Anchor drug, a well-established primary therapeutic agent with known efficacy for specific targets, with Library drug, a supplementary drug to enhance the efficacy of anchor drugs and broaden the therapeutic approach. Our work focused on harnessing regression-based Machine learning (ML) and deep learning (DL) algorithms to develop a structure-activity relationship between the molecular descriptors of drug pairs and their combined biological activity through a QSAR (Quantitative structure-activity relationship) model. 11 popularly known machine learning and deep learning algorithms were used to develop QSAR models. A total of 52 breast cancer cell lines, 25 anchor drugs, and 51 library drugs were considered in developing the QSAR model. It was observed that Deep Neural Networks (DNNs) achieved an impressive R^2^ (Coefficient of Determination) of 0.94, with an RMSE (Root Mean Square Error) value of 0.255, making it the most effective algorithm for developing a structure-activity relationship with strong generalization capabilities. In conclusion, applying combinational therapy alongside ML and DL techniques represents a promising approach to combating breast cancer.

## 1 Introduction

Breast cancer is the most common, lethal, malignant, and highly heterogenic cancer among cancers worldwide (Di Nardo et al., 2022). Information sourced from the North American Association of Central Cancer Registries depicted around 300,590 (2800 in males and 297,790 in females) estimated new cases and 43,700 (530 in males and 43,170 in females) estimated deaths because of breast cancer, making it the leading cancer in estimated new cases and top second leading cancer in estimated deaths for females as shown in Supplementary Table S1 (Siegel et al., 2023). Classified into ductal and lobular carcinoma, treatment strategies vary based on genomic features, including EGFR2 activation and genetic mutations (BRCA1, BRCA2, PIK3A) (Dahan et al., 2023). Therapeutic options, such as radiotherapy, immunotherapy, hormone therapy, chemotherapy, and targeted therapies, are tailored to the patient’s profile (Dahan et al., 2023). Early-stage breast cancer responds to surgery, chemotherapy, and pre-operative neo-adjuvant therapies, enhancing surgical outcomes (Hong and Xu, 2022).

While various therapeutic approaches exist, addressing this complex tumor presents a global challenge for researchers. Combinational chemotherapy, involving the simultaneous administration of two drugs, has been explored to impact tumor progression and metastasis (Farghadani and Naidu, 2022). In a study by Reyhaneh Farghadani et al., in 2022, curcumin was investigated for its ability to enhance the biological activity of existing drugs, leading to reduced tumor size and improved prognosis. *In vitro* studies on breast cancer cell lines revealed increased efficacy of cisplatin, doxorubicin, paclitaxel, and 5-fluorouracil when combined with curcumin (Farghadani and Naidu, 2022). Another avenue explored by Hui-Hui Zhang et al., in 2016 involved the use of metformin to enhance the efficiency of various drugs, including 5-fluorouracil, epirubicin, cyclophosphamide, doxorubicin, and paclitaxel when administered in combination (Iliopoulos et al., 2011; Soo et al., 2015; Zhang and Guo, 2016). Combinational chemotherapy is a promising strategy for breast cancer diagnosis, representing a growing research area in drug discovery and computational biology.

Quantitative Structure-Activity Relationship (QSAR) is a data-driven approach in ligand-based drug discovery. It relies on molecular descriptors—quantitative representations of a molecule’s structure. These descriptors encompass topological, geometric, electronic, and physicochemical characteristics. The primary goal of QSAR is to predict the biological activity of molecules based on these descriptors, providing valuable insights for drug development (Ma et al., 2015). This ligand-based approach aims to correlate the structure of a molecule with its activity, helping filter out inactive molecules and prioritize experiments with selected compounds in early drug development stages (Ma et al., 2015). QSAR models vary based on the molecular descriptors, including 2-dimensional QSAR, 3-dimensional QSAR, and 4-dimensional QSAR (Sippl, 2000; Roy et al., 2015; Bak, 2021; Mishra et al., 2021). Molecular descriptors are the variables that quantitatively represent a molecule, and these can be Topological descriptors, Geometric descriptors, Electronic descriptors, physicochemical descriptors, QSAR descriptors, chemical fingerprints, and Molecular fingerprints (Soares et al., 2022). In the past decade, artificial intelligence, particularly machine learning (ML) and deep learning (DL) has made remarkable strides in drug discovery (Chen et al., 2018; Smith et al., 2018; Lane et al., 2021; Wu et al., 2021). ML and DL, recognized as data-driven approaches, play a significant role in developing Quantitative Structure-Activity Relationship (QSAR) models for various diseases (Sippl, 2000). These models, employed for regression-based predictions of continuous variables like Biological activity (IC_50_ values), leverage machine learning and deep learning methods. Traditional QSAR modeling entails calculating molecular descriptors for each drug molecule, using the generated data to train ML algorithms. The trained algorithms can then predict the biological activity of novel molecules based on structural information. Performance metrics like R^2^ (Coefficient of determination), RMSE (Root Mean Square Error), MSE (Mean Square Error), and Fold Cross-validation scores are assessed for validation in regression-based machine learning predictions (Wu et al., 2021).

QSAR models, integrating Machine Learning (ML) and Deep Learning (DL), demonstrate versatility across diverse diseases. Wu et al. (2021) conducted a comparative study on 16 ML algorithms, identifying rbf-SVM, XGBoost, and rbf-GPR as top performers. Kleandrova et al. (2020) introduced the first cell-based multi-target QSAR model for hepatic carcinoma. Additionally, Alejandro et al. (2020) innovatively applied Perturbation theory-based ML to predict antisarcoma compound activity, leveraging data from assay organisms, cell lines, and target proteins (Cabrera-Andrade et al., 2020). These advancements exemplify the evolving precision and adaptability of ML and DL in shaping effective QSAR models for varied diseases.

Jaaks et al. (2022) conducted a comprehensive investigation to identify effective drug combinations for breast, pancreatic, and colon cancer cell lines. The outcomes of their study were meticulously documented in the GDSC^2^ database. Our approach stands out as unique by leveraging the GDSC^2^ Combinations database, providing distinct insights into the biological activity of anchor drugs, library drugs, and their combinations across breast cancer cell lines. Notably, it is worth mentioning that, at the time of our study, the GDSC^2^ database had not been utilized by any researcher for a QSAR study of this nature. Instead of relying on commercial software, we employed regression-based Machine Learning (ML) and Deep Learning (DL) algorithms to craft a Quantitative Structure-Activity Relationship (QSAR) Model for predicting biological responses. The dataset includes two types of drugs: Anchor drugs and Library drugs. Anchor drugs are well-established medications known for their effectiveness on specific targets, serving as the primary therapeutic agents. On the other hand, Library drugs, also referred to as supplementary or adjunct drugs, are used in conjunction with anchor drugs to enhance their effectiveness. Library drugs are strategically employed to diversify the therapeutic approaches of combination therapy (Jaaks et al., 2022). The dataset provides details on combinational biological activity (Combo IC_50_) values for drug pairs, target pathways, effectiveness values of library drugs, and measures of synergy as indicated by Bliss Emax for anchor drugs and Combo Emax. These attributes collectively offer insights into the combined impact of drugs. For a more in-depth understanding, please refer to Supplementary Table S2.

We calculated molecular descriptors for both the Anchor and Library drugs using the Padelpy library in Python v3.12.0, with Combo IC_50_ as the target variable and other attributes as independent variables. Using regression-based Machine Learning algorithms, we developed a structure-activity relationship to understand interactions and patterns among the drugs’ molecular descriptors and combined biological activity. Comparative regression analysis on test and validation sets evaluated model performance. Notably, our approach of constructing a combinational QSAR model, considering two drugs, is novel in comparison to traditional single-drug QSAR models. Details are provided in the subsequent sections.

## 2 Materials and methods

**FIGURE 1:**
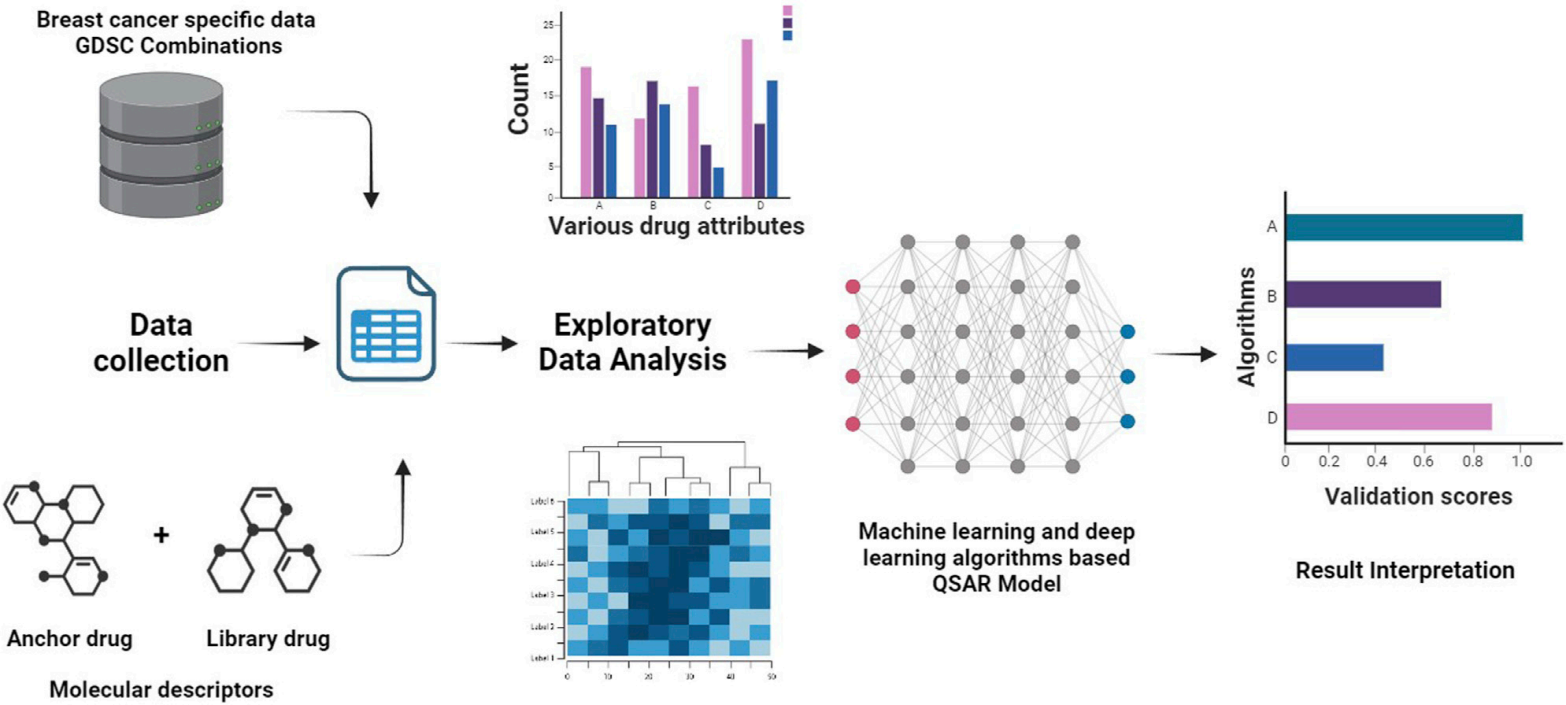
A graphical abstract illustrating the Research.

We utilized the GDSC^2^ (Genomics of Drug Sensitivity in Cancer) combinations database to acquire breast cancer data, followed by a series of preprocessing steps. Regression-based machine learning algorithms, well-known in the field, were then employed to predict Combo IC_50_ values for 52 cancer cell lines. A graphical abstract is represented in Figure 1. We thoroughly validated and documented the model’s performance.

### 2.1 Data collection

The GDSC^2^ database provides breast cancer-specific data and comprises information from 52 cell lines. Molecular Descriptors were calculated using the Padelpy library in Python v3.12.0.

### 2.2 Data pre-processing

**FIGURE 2:**
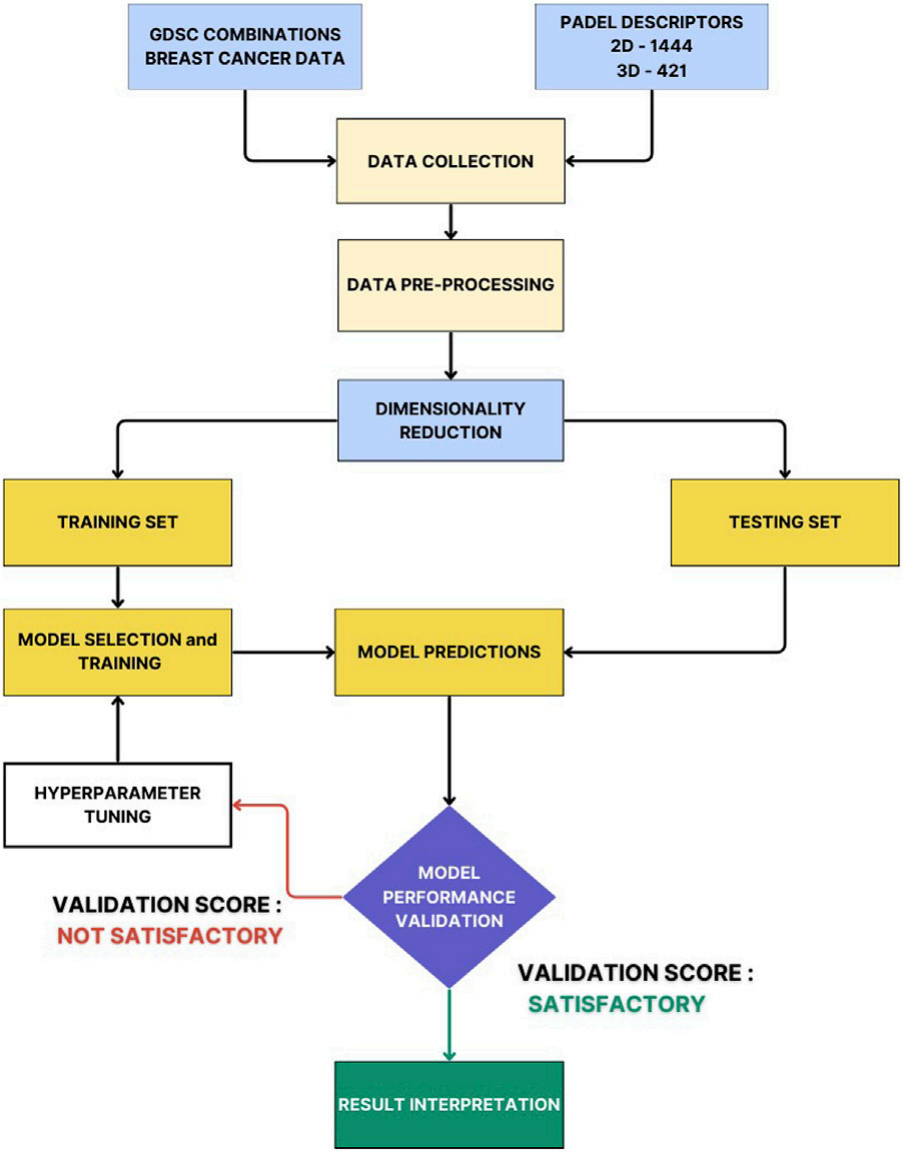
A Complete workflow of developing a combinational QSAR Model using ML and DL.

Figure 2 illustrates the process where Principal Component Analysis (PCA) was applied to reduce dimensionality, effectively minimizing noise and producing a dataset that retains 95% of the explained variance from the initial data. Skewness and kurtosis values were calculated, and outliers were addressed through Boxcox, yeojohnsons, and logarithmic transformations to ensure a normal distribution. Following this, data encoding and standardization were performed using the Scikit-learn library in Python v3.12.0. This preprocessing aimed to facilitate subsequent supervised regression-based machine learning and deep learning predictions.

### 2.3 Supervised machine learning

We employed eleven well-known regression-based machine learning algorithms for QSAR model development, including Random Forest (RF), Extra Gradient Boost (XGB), Ridge Regression, k-Nearest Neighbours (kNN), LASSO Regression, Elastic Net Regression, CART (Classification and Regression Trees), Stochastic Gradient Descent Regressor (SGD), Support Vector Regressor (rbf-SVR), Wider Neural Network (WNN), and Deep Neural Network (DNN) as shown in Table 1. The optimized hyperparameters for each algorithm are outlined in Table 2, and additional details for each algorithm are provided in the Supplementary Data. The preprocessed dataset was partitioned into training, testing, and validation sets in a 60:20:20 ratio using the Scikit-learn library in Python v3.12.0.

**TABLE 1:** Summary of validation metrics calculated for all the ML and DL algorithms employed on the test set.

Algorithm	MAE	RMSE	Explained variance	R - Square	MSE
RF	0.614	0.95	0.88	0.88	0.902
XGB	0.464	0.27	0.92	0.92	0.072
LASSO	0.7856	0.78	0.81	0.81	0.608
ELASTIC NET	1.236	1.01	0.79	0.79	1.02
k-NN	0.745	0.929	0.77	0.77	0.863
SGD-Regression	0.6732	0.503	0.82	0.82	0.253
CART	0.446	0.39	0.83	0.83	0.152
SVR-rbf	0.326	0.28	0.91	0.91	0.078
Ridge	0.4587	0.478	0.74	0.74	0.228
DNN	0.248	0.255	0.94	0.94	0.065
Wide-NN	0.458	0.365	0.86	0.86	0.133

**TABLE 2:** Summary of the optimized hyperparameters of various ML and DL algorithms.

S. No	Algorithm	Hyperparameters
1	DNN	Input layer with 2516 nodes, Five Hidden layers with 500, 250, 125, and 32 nodes, Activation function = ‘ReLu’, Optimizer = SGD, Learning rate = 0.001, Loss = MSE, Epochs = 100, Batch size = 64, Patience limit = 10, Validation split = 0.25
2	WNN	Input layer with 2516 nodes, Two Hidden layers with 3000 and 2000 nodes, Activation function = ‘ReLu’, Optimizer = Adam, Learning rate = 0.001, Loss = mean square error, Epochs = 100, Batch size = 64, Patience limit (early stopping protocol) = 10, Validation split = 0.25
3	XGB	Maximum depth of 1, Maximum features of 8, Learning rate of 0.08, 10,000 estimators, Loss = mean absolute error
4	SVR - rbf	Kernel = radial basis function, Epsilon value of 0.9, Gamma was set to scale
5	RF	10 leaf nodes, 1000 decision trees, Minimum sample split of 5, Minimum sample leaves of 2, Maximum depth of 10
6	KNN	Nearest neighbours count (k) = 5, Uniform weight, Euclidian distance metrics, Leaf size = 10
7	Ridge	Alpha = 20
8	SGD	L2 regularization with (Lambda) = 0.7, Learning rate = 0.001, Number of iterations with no improvement in validation score = 250, Maximum number of epochs = 1000
9	LASSO	Alpha = 0.5
10	Elastic Net	Alpha = 0.5, Lambda = 0.6
11	CART	Maximum depth of tree = 50, Minimum sample split = 10, Sample leaf count = 5, Number of features considered at each split to none, Maximum number of leaf nodes to 100

### 2.4 Model performance evaluation

We validated the model’s performance and interpreted predictions by assessing key validation scores, including RMSE (Root Mean Square Error), MSE (Mean Square Error), MAE (Mean Absolute Error), R^2^ (Coefficient of Determination), Explained variance, and employing an eight-fold cross-validation approach. The SHAP (Shapley Additive Explanations) module in Python v3.12.0 was also utilized for further interpretability (Wu et al., 2021). Evaluation of both test and validation datasets using these metrics ensures the model’s accurate predictions and a well-fitted performance.

## 3 Results

The dataset underwent thorough preprocessing and detailed exploratory data analysis. To elucidate the influence of target pathways across the 52 cell lines under study, we generated heatmaps depicting the frequency distribution of anchor drug and library drug target pathways as shown in Supplementary Figures S1, S2 respectively. Additionally, a comprehensive correlation heat map is showcased in Supplementary Figure S3. To reduce dimensionality while preserving crucial information, Principal Component Analysis was strategically employed. Initially, with 1444 2D descriptors and 421 3D descriptors, the dataset underwent meticulous reduction. Dimensionality was refined to 1000 2D descriptors 376 3D descriptors for anchor drugs, and 766 2D descriptors and 358 3D descriptors for library drugs. Corresponding Scree plots for dimensionality reduction were featured in Supplementary Figure S4.

Combinational QSAR models were established by employing 11 commonly used regression algorithms. To assess prediction performance, key metrics such as R^2^, RMSE, MSE, MAE, Explained Variance and an eight-fold cross-validation were applied to both the Test and Validation sets. Emphasizing the significance of R^2^ and RMSE for evaluating the goodness of fit and the average magnitude of errors between predicted and actual values, these metrics were prioritized for comparison with similar QSAR modeling studies.

**FIGURE 3:**
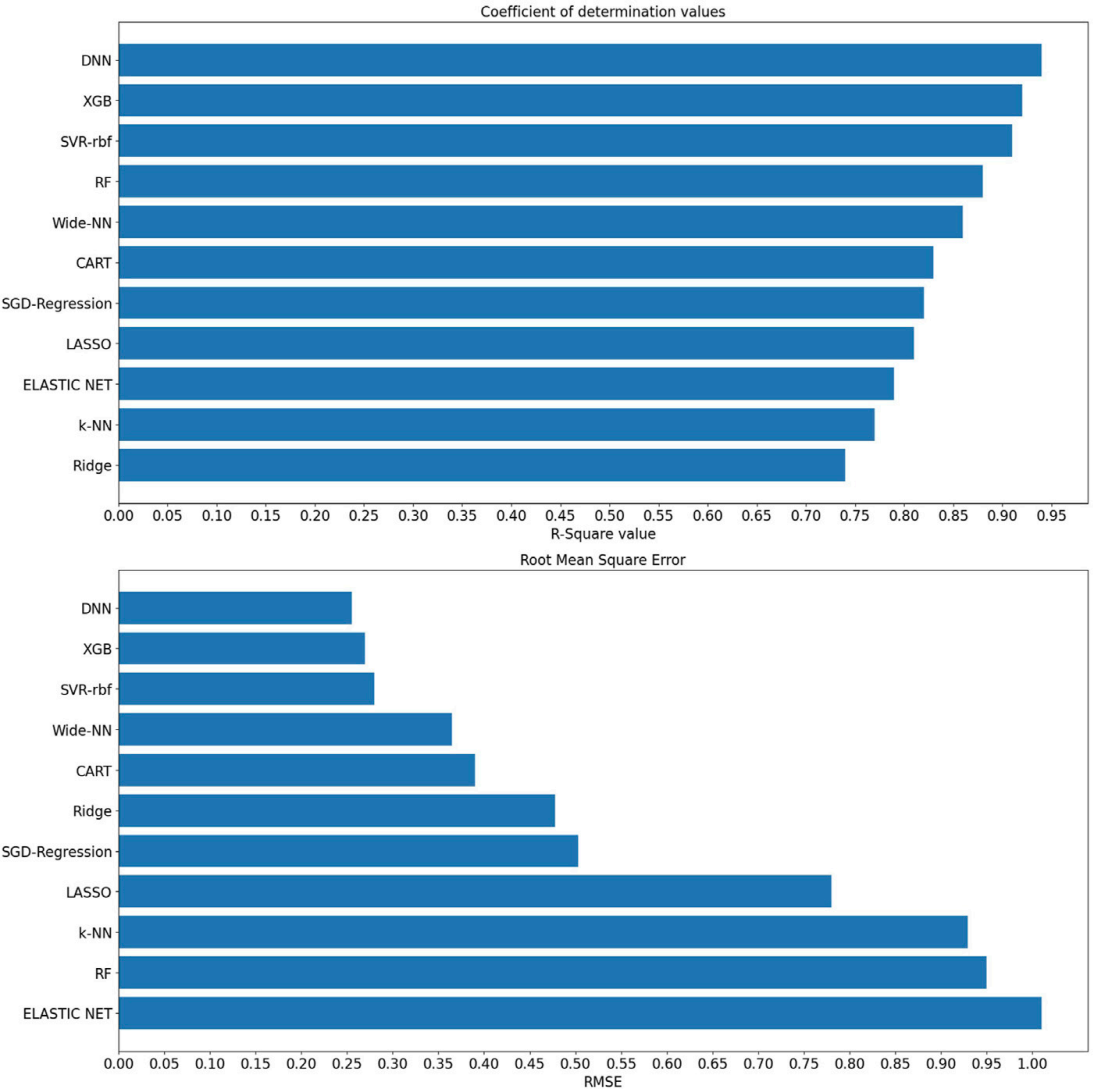
R^2^ and RMSE values of various machine learning algorithms on the test set.

**FIGURE 4:**
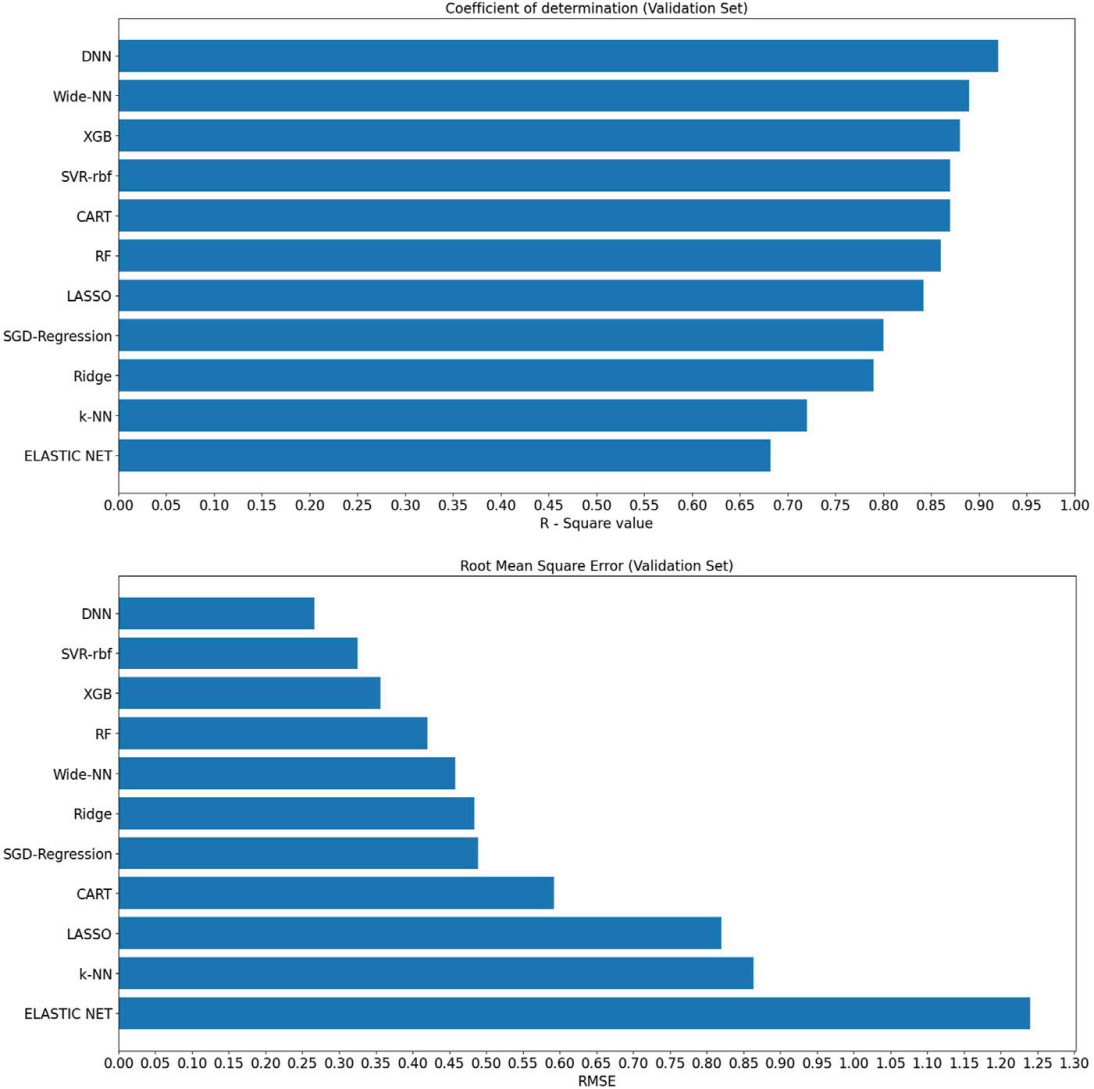
R^2^ and RMSE values of various machine learning algorithms on the validation set.

Upon analyzing the test dataset results, the DNN algorithm demonstrated notable performance with an impressive R^2^ of 0.94 and an associated RMSE value of 0.255, underscoring its ability to make accurate predictions. Following closely were Algorithm XGB and rbf-SVR, achieving R^2^ values of 0.92 and 0.91, along with RMSE values of 0.278 and 0.289, respectively, as illustrated in Figure 3. Transitioning to the validation set, the DNN algorithm continued to excel with an R^2^ value of 0.92, indicative of an excellent model fit. Wider-NN (Neural network) and XGB (Extra Gradient Boost) algorithms followed with R^2^ values of 0.896 and 0.885, respectively, revealing promising results and a commendable fit, as depicted in Figure 4. Nevertheless, it is essential to note that algorithms Ridge Regression, Elastic Net, and KNN (K-Nearest Neighbours) struggled to perform well in both validation and test datasets. Their lower R^2^ values of 0.74, 0.79, and 0.77 for the test set and 0.79, 0.682, and 0.72 for the validation set and higher RMSE values of 0.478, 1.01, 0.929 on the test set and 0.483, 1.24, and 0.864 on the validation set suggest a sub-optimal prediction. Validation metrics considered for all 11 algorithms were tabulated in the Table. 1.

We have employed the SHAP (SHapely Additive explanations) approach to gain insight into the top 20 essential attributes and their impact on driving the predictions. SHAP (Shapley Additive explanations) scores reveal the key attributes that have positive (positive SHAP scores) and negative (Negative SHAP scores) impacts on the model predictions. Accordingly, those descriptors can be considered while developing similar QSAR models. The top 20 attributes with positive, negative, and overall SHAP values and their magnitude in contributing to model predictions are shown in Supplementary Figures S5–S7. Based on our analysis, as shown in Supplementary Table S3, it became apparent that descriptors related to the electronic state possess more impact as crucial attributes in the development of a combinational QSAR model.

**FIGURE 5:**
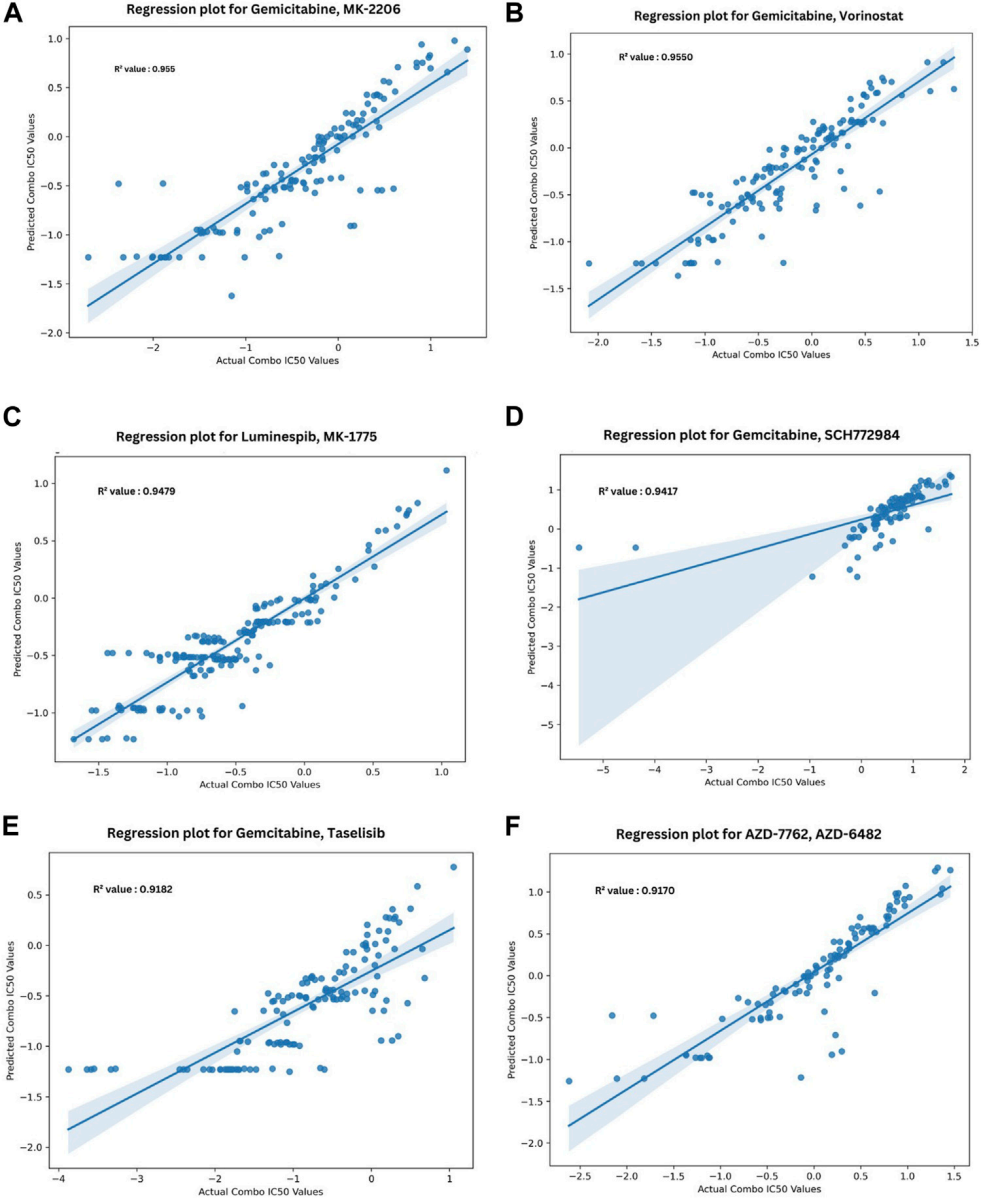
Top six regression plots for Anchor—Library drug combinations obtained from DNN-based QSAR model. **(A–F)** Represent the drug combinations Gemcitabine—MK-226, Gemcitabine– Vorinostat, Luminespib–MK-1775, Gemcitabine–SCH772985, Gemicitabine–Taselisib, AZD7762—AZD6482, Gemicitabine–Taselisib, AZD7762—AZD6482 respectively (Negative IC_50_ values are presented due to logarithmic transformation during the preprocessing stage, enhancing data representation and clarity in the graph).

**FIGURE 6:**
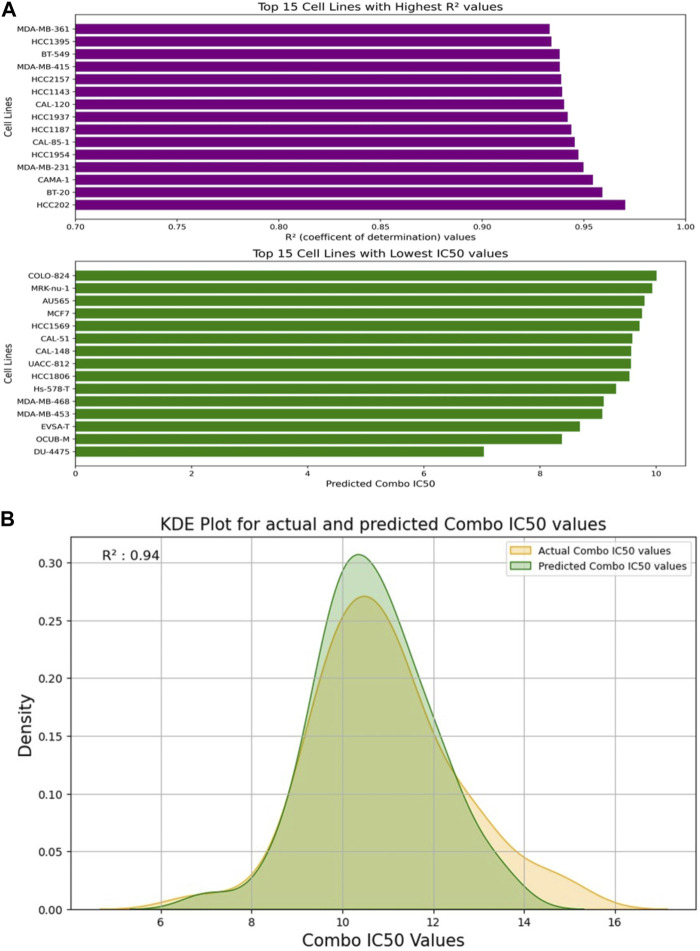
**(A)** Visualization of the Top 15 Cell Lines: Highest R^2^ Values and Lowest IC50 Values from the Test Set. **(B)** Kernel Density Estimation (KDE) Plot: Distribution of Actual Combo IC_50_ Values vs. Predicted Combo IC_50_ Values by the DNN-QSAR Model.

Figure 5 depicts the validation set results of the DNN-based QSAR model, highlighting impressive R2 values for the top six specific drug combinations selected from a pool of 1200 possible drug combinations in the dataset. These combinations include Gemicitabine-MK-226, Gemicitabine-Vorinostat, Luminespib-MK-1775, Gemicitabine-SCH772985, Gemicitabine-Taselisib, and AZD7762-AZD6482. MDA-MB-361, HCC1395, and BT-549 cell lines demonstrated top R^2^ values, while COLO-824, MRK-nu-1, and AU565 exhibited a low IC_50_ value as shown in Figure 6A. Figure 6B visually compares the distribution of Actual Combo IC_50_ Values and Predicted Combo IC_50_ Values, affirming the QSAR model’s reliability. Additionally, Supplementary Figure S8 presents the chemical structures of the highlighted drug combinations (from Figure 5) with corresponding target and pathway information. These comprehensive findings support the QSAR model’s potential in virtual screening of unknown molecules and drug repurposing, providing a valuable contribution to drug discovery.

## 4 Discussion

Jaaks et al., 2022 extensively explored effective drug combinations for breast, pancreatic, and colon cancer cell lines, documented in the GDSC^2^ database. Our unique approach leverages the GDSC^2^ Combinations database, providing distinctive insights into the biological activity of anchor drugs, library drugs, and their combinations in breast cancer cell lines. Rather than relying on commercial software, we employed regression-based Machine Learning (ML) and Deep Learning (DL) algorithms to construct a novel Quantitative Structure-Activity Relationship (QSAR) Model for predicting biological responses. This pioneering model integrates the intricate interplay between anchor and library drugs, diverging from conventional QSAR methodologies (Kausar and Falcao, 2018) and multimodal deep learning techniques (Vale-Silva and Rohr, 2021; Boehm et al., 2022). 11 distinct ML and DL algorithms sourced from GDSC^2^ combinations, underwent rigorous performance validations to ensure robust predictive capabilities. This innovative approach broadens the scope of QSAR modeling and contributes to the understanding of drug interactions in cancer biology.

With an R^2^ value of 0.94 and RMSE of 0.255 our Combinational QSAR model outperformed existing models, which typically considered single drugs and genomic parameters (Speck-Planche et al., 2011). A comparative study by Junshui et al., in 2014, evaluating DNN and other ML algorithms for QSAR models, achieved a top R^2^ value of 0.82 (Ma et al., 2015). Guided by Wu et al. a comprehensive assessment of 16 ML models from various datasets in 2021, our study’s framework was established (Wu et al., 2021). By incorporating a broader spectrum of cancer cell lines, our model enhances its generalizability in predicting drug responses across diverse cancer types. Unlike other studies concentrating on the biological activity of a single molecule, our focus was on the combinational activity of two molecules. Through an unconventional yet strategic comparison, our developed model distinctly showcases superior performance, addressing a crucial research gap by providing a robust machine learning-based QSAR model for predicting combinational drug responses.

Our study employs the SHAP approach to identify crucial chemical moieties for anti-cancer activity, such as the Geary Autocorrelation at Lag 5 weighted by I state, Maximum Atom-type E-State with a focus on oxygen (-O), and the Normalized Randic-like Eigenvector-based Index from the Detour Matrix. Refer to Supplementary Table S3. These descriptors align well with established biological principles. For instance, the Geary Autocorrelation provides insights into spatial electronic patterns influencing interactions with specific cellular targets in cancer progression, and the Maximum Atom-type E-State’s emphasis on electron-donating capability may impact the modulation of key enzymes or receptors in anticancer pathways. The Normalized Randic-like Eigenvector-based Index reflects molecular topology and connectivity, influencing interactions with crucial cellular components involved in cancer-related processes. These interpretations offer a nuanced understanding of the biological relevance of these descriptors. The chemical molecules structurally similar as mentioned in Figure 5 are proposed to possess anti-cancer activity by the QSAR model. Additionally, our versatile combinational QSAR model, with an R2 value of 0.94 and RMSE of 0.255, revealed top drug combinations with the highest R^2^ values targeting key pathways like MTOR/PI3K signaling, chromatin histone acetylation, DNA replication, ERK/MAPK signaling, protein stability and degradation, and cell cycle regulation. These combinations mentioned in Figure 5 and Supplementary Figure S8 were validated and demonstrated significant effects on breast cancer cell progression through modulation of these critical pathways (Guo et al., 2018; Guo et al., 2020; Miricescu et al., 2020).

Careful consideration of procurement expenses for cell lines and pharmaceuticals, along with associated maintenance costs in research laboratories, is essential for researchers. The financial and temporal complexities of this process underscore its inherent challenges. In the domains of anticancer drug development and therapeutic strategy research, predictive models are crucial. They prove invaluable in early-stage drug development by identifying non-responsive or less responsive drugs and optimizing resource allocation in terms of finances, time, and human efforts. Conventional Quantitative Structure-Activity Relationship (QSAR) models have their merits in predicting drug responses, yet the proposed combinational QSAR models offer a distinct advantage. They enhance our ability to predict the activity of unknown combinations and forecast responses in drug repurposing scenarios. While neither conventional nor combinational QSAR models replace actual wet lab *in vitro* and *in vivo* studies, they play a pivotal role in complementing these studies, effectively addressing research gaps, and providing valuable insights.

In conclusion, our endeavor in developing combinational QSAR models has been shaped by the observation that the dataset within the repository, while valuable, is comparatively limited when juxtaposed with other extensive drug databases. Acknowledging the intrinsic strength of a data-driven approach, we recognize the potential for enhanced pattern identification and increased accuracy in predictions with a more expansive dataset. Looking forward, the future trajectory of this approach holds promise in the integration of genomic, proteomic, and transcriptomic data through multimodal deep learning methodologies. This expanded integration seeks to discern intricate patterns among omics data and drug response data, thereby enabling more efficient prediction of outcomes. Furthermore, incorporating various stereoisomers and conformers derived from existing molecules can broaden the applicability domain of our models. Leveraging the capabilities of machine learning and deep learning in the development of versatile QSAR models, encompassing omics data, drug response data, and even image data such as tissue section images, stands as a dynamic strategy. This strategic amalgamation empowers us to efficiently screen the ever-expanding pool of drug molecules, swiftly eliminating non-potential candidates in the early stages of drug discovery, constituting a significant advancement in the field.

## Data Availability

Publicly available datasets were analyzed in this study. This data can be found here: https://gdsc-combinations.depmap.sanger.ac.uk/ GDSC (Genomics of drug sensitivity in cancer)—Combinations.
