# Supplementary material for: A breast cancer-specific combinational QSAR model development using machine learning and deep learning approaches
Source: Front Bioinform. 2024 Jan 15;3:1328262. doi: 10.3389/fbinf.2023.1328262 (PMC10822965; doi:10.3389/fbinf.2023.1328262)
Supplement: Supplementary file 1 [file DataSheet1.pdf]

Supplementary Material

1 Supplementary Data

Random Forest (RF): Random forest was a widely used ensemble algorithm for regression tasks in drug discovery and QSAR modeling. It is based on the concept of decision trees and combines the output of the decision trees to predict an overall output, improving the accuracy and reducing overfitting. It performs bootstrapping, randomly selecting subsets from drug response data to create multiple subsets, and multiple decision trees handle these, each handling one (1). We have considered ten leaf nodes, 1000 decision trees, a minimum sample split of 5, minimum sample leaves of 2, and a maximum depth of 10 as optimal hyperparameters to develop a combinational QSAR model.

XGBoost (XGB): Extreme gradient boosting is a robust ensemble machine learning algorithm used extensively in regression tasks. An ensemble of decision trees starts with initial predictions, calculating initial residues, and uses regularized trees to reduce overfitting. New predictions were calculated by adding the output of the current tree to the previous predictions. It updates the residuals based on the difference between the actual values and current predictions, and this process will be iterated based on the number of estimators given (2). XGB algorithm was trained with a maximum depth of 1, maximum features of 8, a learning rate of 0.08, several estimators of 10000 estimators, and loss was assigned as absolute error.

Ridge Regression: Ridge regression is a linear Machine learning algorithm widely used to predict response variables based on a set of independent variables that were highly correlated. It finds the coefficient of the independent variables in a linear equation that best predicts the response variable and introduces L2 regularization, which adds a penalty term (alpha) to the equation and prevents overfitting by discouraging significant coefficients for individual descriptors and regularization parameters controlling the strength of the penalty(3). The alpha value of 20 was used while training the model.

KNN(k-NearestNeighbors): KNN is a nonlinear machine algorithm that predicts data points based on their proximity to other data points in feature space. KNN made predictions based on the nearest neighbors of a data point in feature space and was chosen based on various hyperparameters such as distance metrics, weights, and neighbor count. The average of the values of nearest neighbors was considered for regression-based tasks (3). The KNN algorithm was trained with five nearest neighbors count, uniform weight, and Euclidian distance metrics with a leaf size of 10.

LASSO: We have leveraged LASSO regression, a linear algorithm that had an impressive role in handling high-dimensional data and extracting essential relationships between molecular descriptors and biological responses. LASSO provides L1 regularization, adding a penalty value alpha, which drives some coefficient estimates to precisely zero in the linear regression equation, identifying the most relevant features for predicting biological response. We have performed cross-validation experiments systematically to identify the optimal level of regularization (3). An alpha value of 0.5 was used in LASSO regression to train the model with the drug response data.

Elastic Net Regression: Elastic Net is a linear regression model that leverages the L1 (alpha) and L2 (lambda) regularization techniques to establish a relationship between molecular descriptors and the biological activity of the drugs by simultaneously selecting essential variables from the training dataset (L1) and reducing multicollinearity (L2)(4). By experimenting with various alpha and lambda values, we have identified optimal regularization values alpha as 0.5 and lambda as 0.6 and selection as random for biological activity prediction.

CART (Classification and Regression Trees): It is a nonlinear decision tree-based machine learning algorithm used for regression tasks. CART's strength lies in capturing the relationship between molecular descriptors and the biological activity of drugs through recursively partitioning the feature space into homogenous regions. It involves the construction of decision trees, with each node representing a feature and selecting the features that maximize the reduction in variance(2,4). Hyperparameter tuning was performed to identify the optimal parameters: maximum depth of a tree to 50, minimum samples split of 10, minimum sample leaf count to 5, number of features to consider at each split, and maximum number of leaf nodes to 100.

Stochastic Gradient Descent Regressor (SGD) : SGD is a linear machine learning algorithm potentially used in QSAR tasks to efficiently capture the relationship between the molecular descriptors and biological activity of the drugs by iteratively updating the model coefficients to minimize the loss function (mean square error). Models coefficients were optimized by SGD regressor on the chosen loss function. Coefficients were updated with smaller learning rates to minimize the loss, making it suitable for large datasets(3). A series of experiments were done to identify optimal hyperparameters for our particular dataset where the number of iterations with no improvement in validation score was set to 250, L2 regularization was applied with lambda value of 0.7, learning rate of 0.001 and maximum number of epochs as 10000.

Support vector regressor (rbf- SVR): rbf-SVR is a machine learning algorithm that potentially captures nonlinear interactions between molecular descriptors and biological activity. The C parameter in the algorithm handles the tradeoff between low training error and low testing error, thus preventing overfitting(4,5). Cross-validation experiments were done by tuning various hyperparameters and identifying optimal parameters. The kernel was selected as 'radial basis function', epsilon, which implies the control of error tolerated in regression predictions as 0.9, and gamma that defines the decision boundary's shape was set as 'scale'.

Wider Neural Network: QSAR model development and machine learning algorithms also used deep learning-based neural networks. Neural networks were the robust algorithms to predict regression-based tasks(5). A more comprehensive Neural network (fewer hidden layers and more neurons in each layer) was employed with one input layer consisting of 2516 units, which served as an entry point for molecular descriptor data. A rectified Linear unit activation function was used, and two hidden layers with 3000 and 2000 units, respectively, were used. Their primary purpose is to learn and represent complex patterns and relationships between molecular descriptors and biological activity, Rectified Linear unit activation function, and a single output layer with one unit responsible for producing the quantitative predictions of the biological activity and linear activation function to ensure the output values were continuous and regression-based task.

Hyperparameter tuning was performed to identify the optimal parameters for better regression-based predictions, and the learning rate was set to 0.001 to ensure a stable convergence and control the step size during the optimization process, Adam optimizer was used to minimize the loss (mean squared

error), training was conducted for 50 epochs with a batch size of 64, early stopping protocol was employed to prevent overfitting with patience limit of 10. In summary, hyperparameters of the neural network were carefully selected and tuned to maintain a balance between model complexity and predictive performance

Deep Neural Network: In a revised QSAR study, we have changed the architecture of the neural networks, developing a deep neural network with an input layer consisting of 2516 nodes, a Rectified linear unit activating function, five hidden layers with 500, 250, 125, 64, 32 nodes respectively and Rectified Linear unit activation function and the output layer consists a single unit responsible for quantitative prediction of continuous variables which were suitable for regression task. Hyperparameter tuning was done to identify optimal parameters, which were a learning rate of 0.001, a momentum of 0.9, Stochastic gradient descent was used as an optimizer, mean squared error was considered for the loss function, epochs were 50, batch size was 64, validation split was set to 0.25(5)

2 Supplementary Figures and Tables

2.1 Supplementary Figures

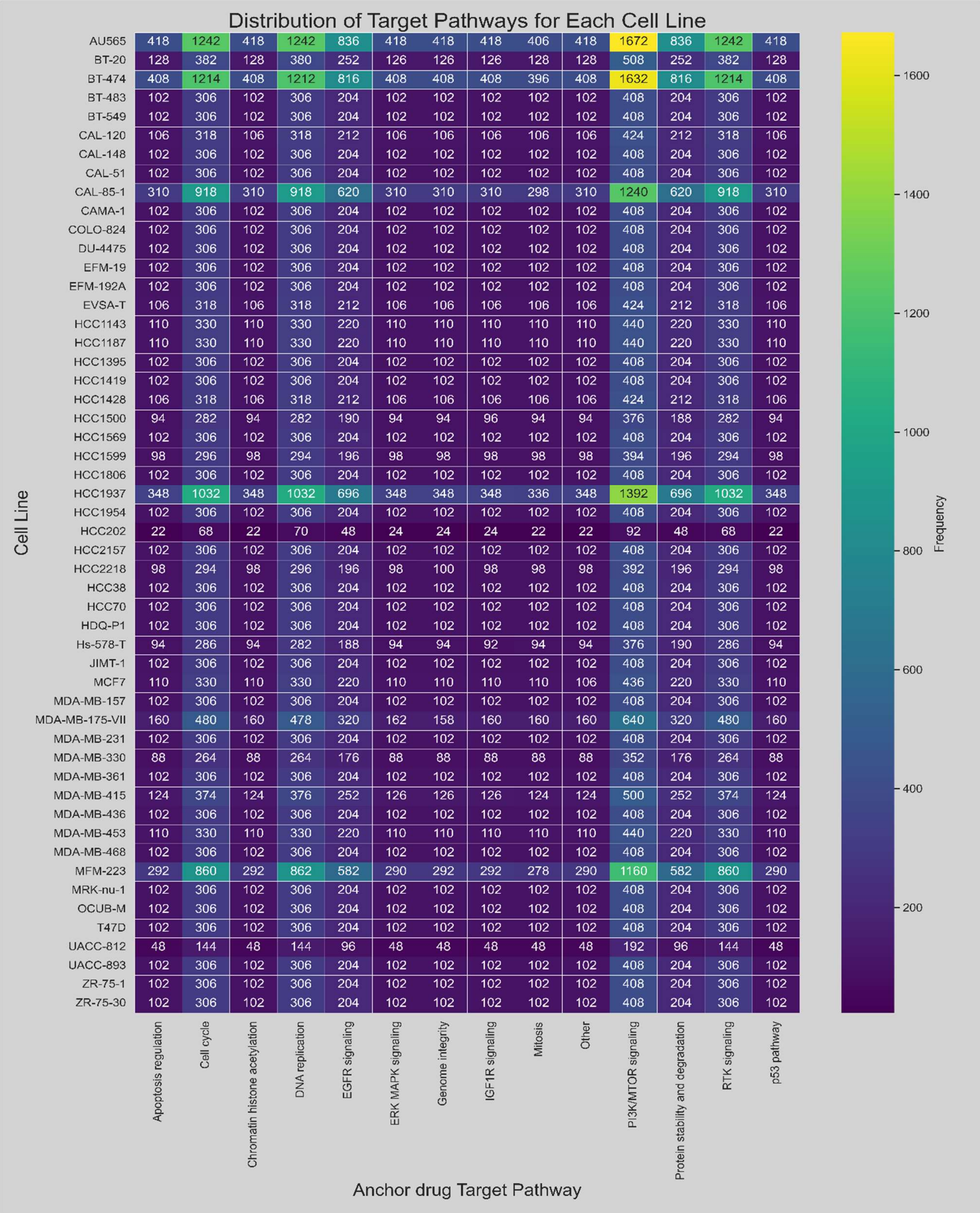

Figure S1 : Heatmap representing the distribution of anchor drug target pathways in various cell lines

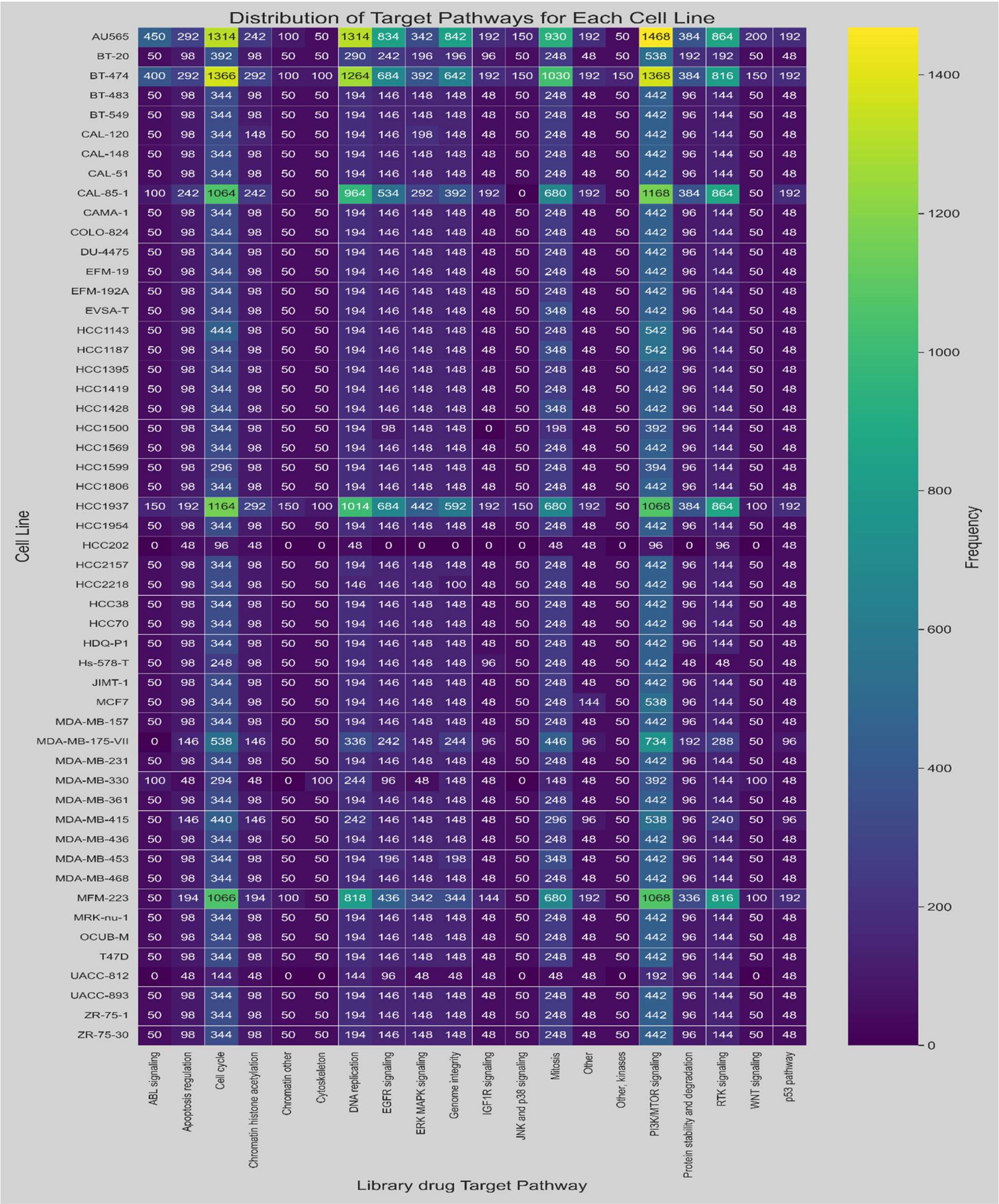

Figure S2: Heatmap representing the distribution of Library drug target pathways in various cell lines

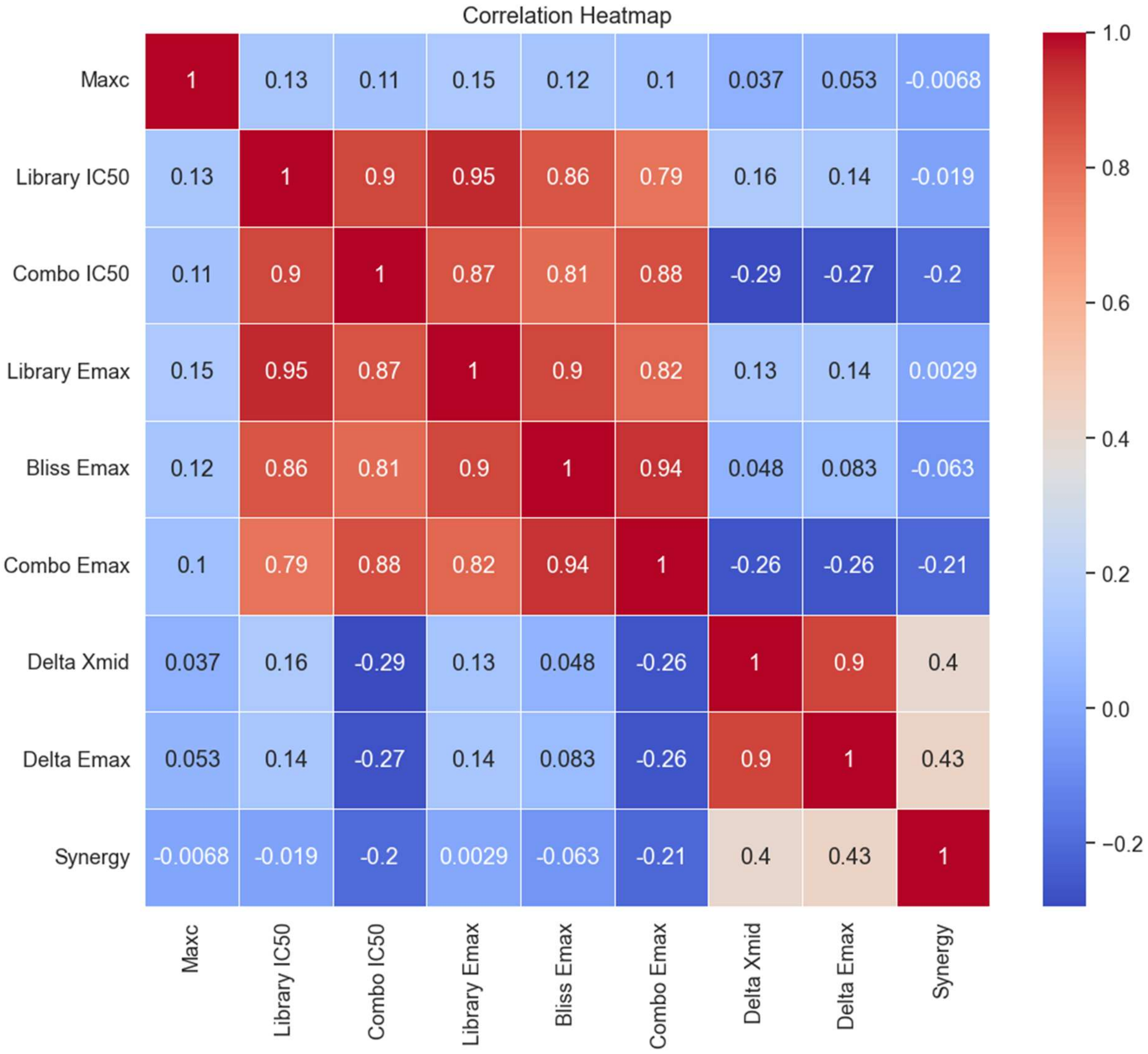

Figure S3: Heatmap representing the correlation among the continuous attributes present in the GDSC combinations dataset

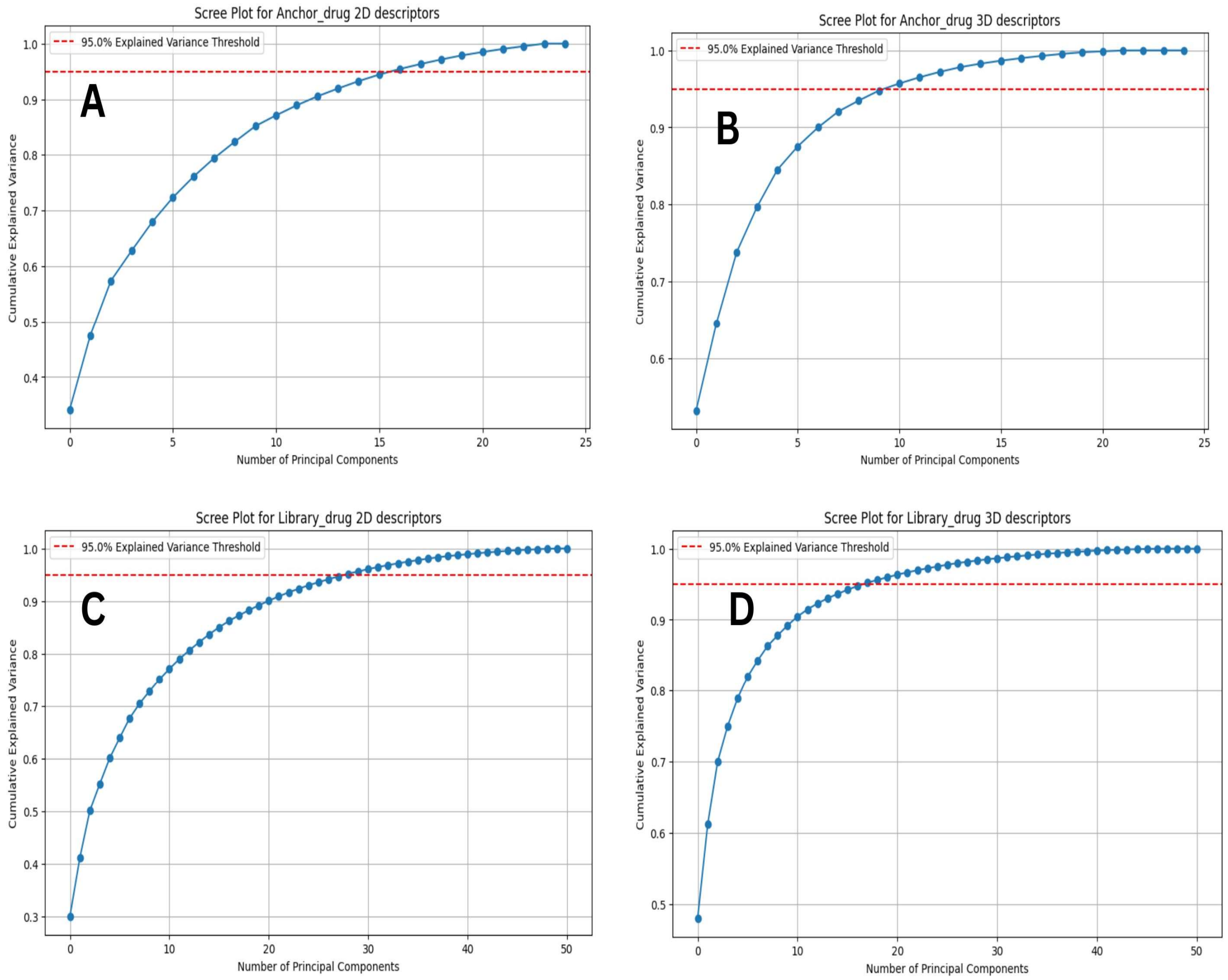

Figure S4: Scree plots representing dimensionality reduction using principal component analysis at 95% explained variance threshold level. A and B – represent the scree plots for 2-dimensional and 3-dimensional descriptors of anchor drugs, and C and D - represent library drugs, respectively.

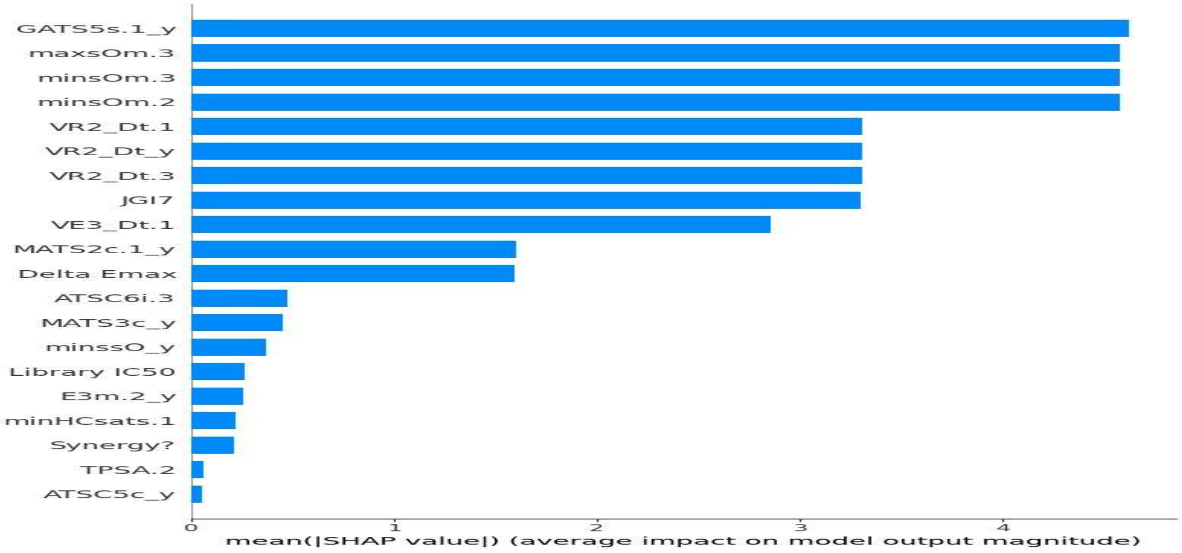

Figure S5: Top 20 attributes from the processed dataset positively impacting model predictions. (Vertical axis represents the molecular descriptors from the dataset and horizontal axis represents the average impact of each descriptor on models output magnitude)

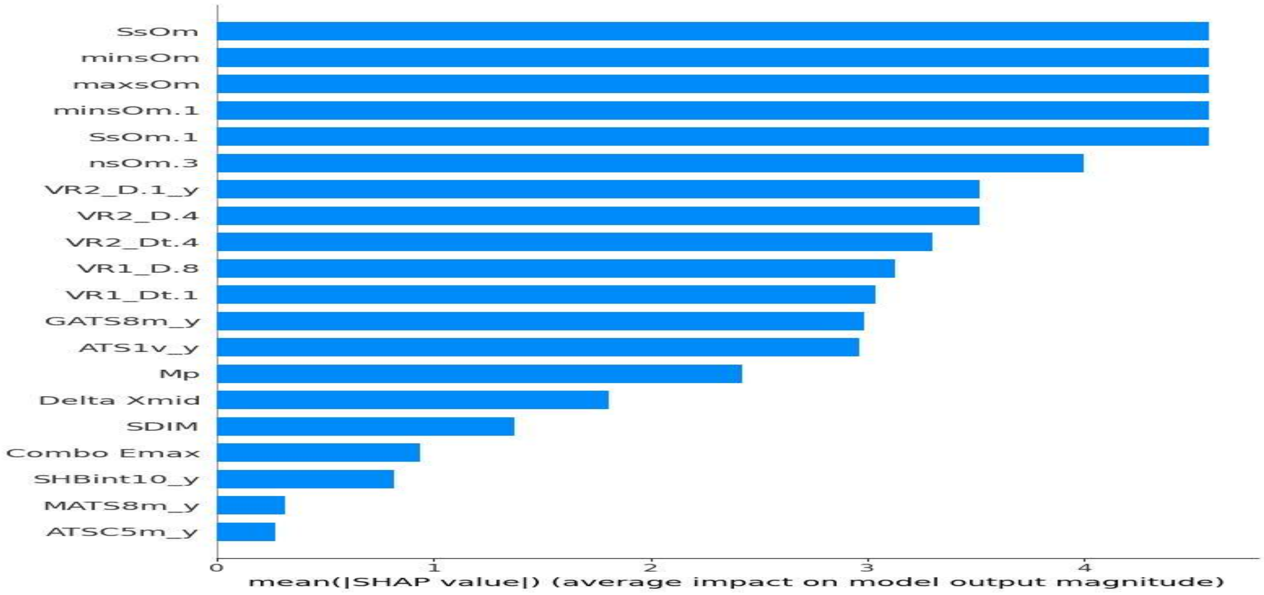

Figure S6: Top 20 attributes from the processed dataset harming model predictions. (Vertical axis represents the molecular descriptors from the dataset and horizontal axis represents the average impact of each descriptor on models output magnitude)

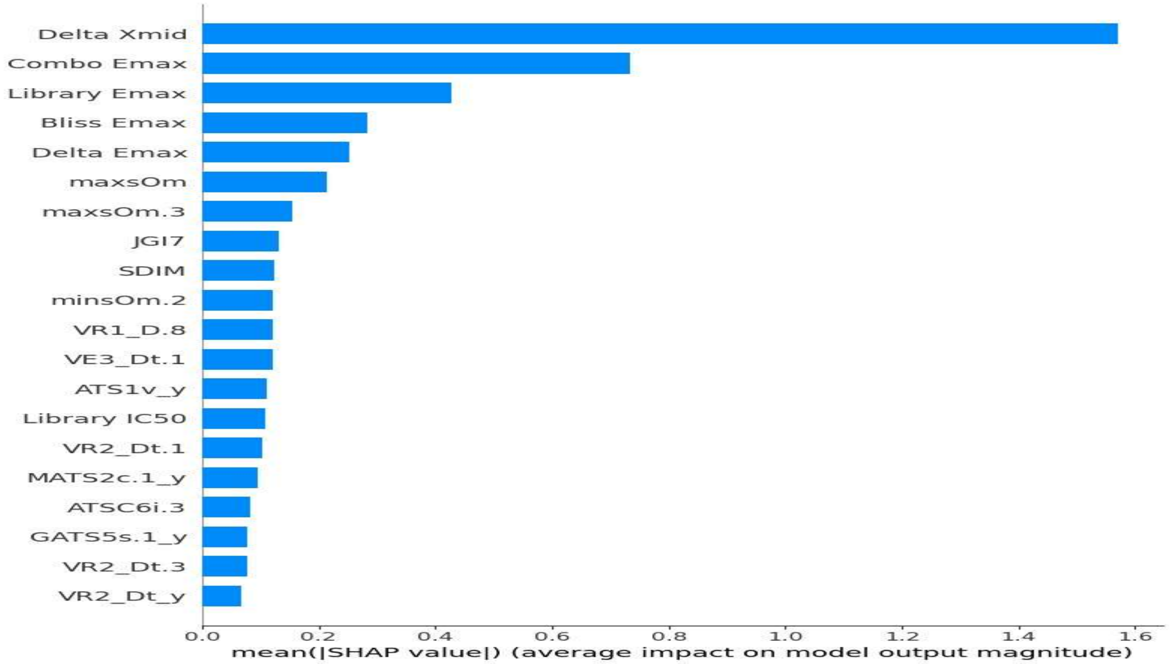

Figure S7: Top 20 attributes with greater importance in model prediction performance. (Vertical axis represents the molecular descriptors from the dataset and horizontal axis represents the average impact of each descriptor on models output magnitude)

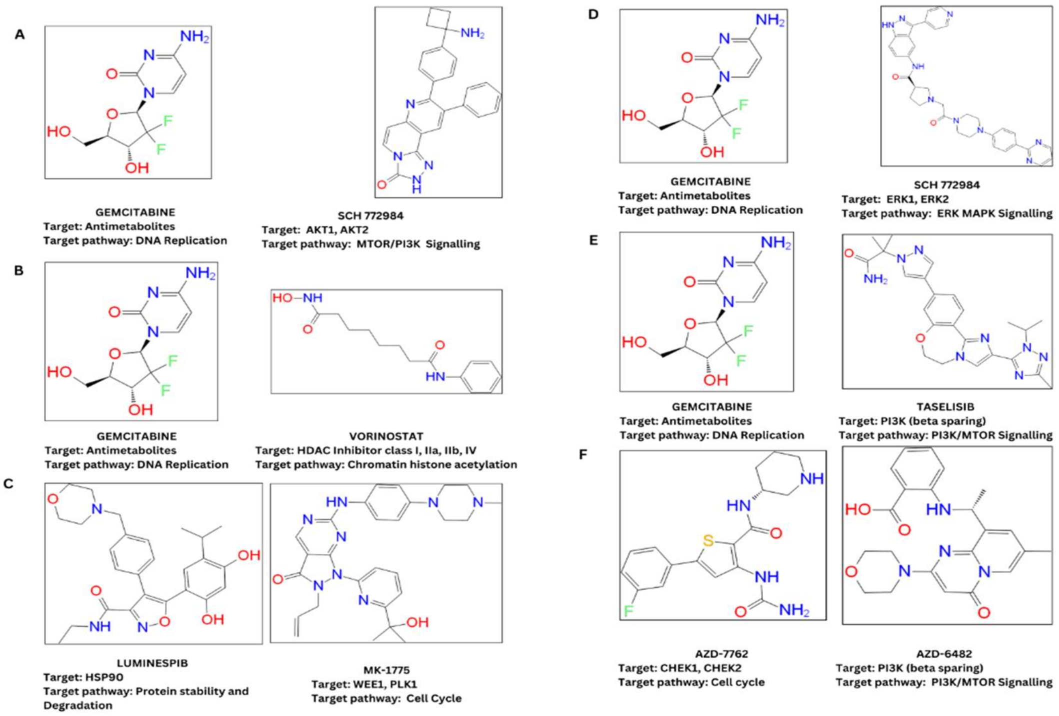

Figure S8: Chemical structures of the top 6 drug combinations and their respective targets and target pathways in cancer cell lines from the validation set.

2.2 Tables

Table S1: Top 10 leading cancers with their respective estimated deaths and estimated new cases percentages (6).

Estimated New Cases

Males			Females		
Prostate	288,300	29%	Breast	297,790	31%
Lung & bronchus	117,550	12%	Lung & bronchus	120,790	13%
Colon & rectum	81,860	8%	Colon & rectum	71,160	8%
Urinary bladder	62,420	6%	Uterine corpus	66,200	7%
Melanoma of the skin	58,120	6%	Melanoma of the skin	39,490	4%
Kidney & renal pelvis	52,360	5%	Non-Hodgkin lymphoma	35,670	4%
Non-Hodgkin lymphoma	44,880	4%	Thyroid	31,180	3%
Oral cavity & pharynx	39,290	4%	Pancreas	30,920	3%
Leukemia	35,670	4%	Kidney & renal pelvis	29,440	3%
Pancreas	33,130	3%	Leukemia	23,940	3%
**All Sites**	**1,010,310**	**100%**	**All Sites**	**948,000**	**100%**

Estimated Deaths

Males			Females		
Lung & bronchus	67,160	21%	Lung & bronchus	59,910	21%
Prostate	34,700	11%	Breast	43,170	15%
Colon & rectum	28,470	9%	Colon & rectum	24,080	8%
Pancreas	26,620	8%	Pancreas	23,930	8%
Liver & intrahepatic bile duct	19,000	6%	Ovary	13,270	5%
Leukemia	13,900	4%	Uterine corpus	13,030	5%
Esophagus	12,920	4%	Liver & intrahepatic bile duct	10,380	4%
Urinary bladder	12,160	4%	Leukemia	9,810	3%
Non-Hodgkin lymphoma	11,780	4%	Non-Hodgkin lymphoma	8,400	3%
Brain & other nervous system	11,020	3%	Brain & other nervous system	7,970	3%
**All Sites**	**322,080**	**100%**	**All Sites**	**287,740**	**100%**

Table S2: Summary of the data sourced from GDSC[2] dataset

Type of cancer	Breast cancer
Number of cell lines	52
Number of Anchor drugs	25
Number of Library Drugs	51
Total number of combinations	1200
Total number of entries in the dataset	163471

Table S3: Drug attributes with crucial contributions to model predictions positively and negatively according to SHAP scores.

S.No	Type of impact on model prediction	Symbol	Name	Description
**1.**	Positive	GATS5s.1_y	Geary autocorrelation at lag5 weighted by I state	It reflects how an atom's property is related to another atom's property, which is five bonds away.
**2.**	Positive	maxsOm.3 and minsOm.3	Maximum atom – type E – State: -O-	These values reflect the molecules with oxygen atoms having the highest and lowest electronic states.
**3.**	Positive	VR2_Dt	Normalized Randic-like eigenvector-based index from detour matrix	It is a complex descriptor that quantifies certain structural features within molecules by analyzing the detour matrix, a mathematical representation of molecular graphs
**4.**	Negative	SsOm	The sum of atom-type E-State: -O-	It indicates the cumulative electronic properties of oxygen atoms within a molecule.
**5.**	Negative	minsOm	Minimum atom-type E-State: -O-	It indicates the lowest electronic state value among the oxygen atoms in a molecule
**6.**	Negative	maxsOm	Maximum atom – type E – State: -O- (Library drug)	These values reflect the molecules with oxygen atoms having the lowest electronic state.

3 References:

1. Chen G, He H, Zhao L, Chen KB, Li S, Chen CYC. Adaptive boost approach for possible leads of triple-negative breast cancer. Chemometrics and Intelligent Laboratory Systems. 2022 Dec 15;231.
2. Kaur G, Gupta R, Hooda N, Gupta NR. Machine Learning Techniques and Breast Cancer Prediction: A Review. Vol. 125, WSireless Personal Communications. Springer; 2022. p. 2537–64.
3. Ponzoni I, Sebastián-Pérez V, Requena-Triguero C, Roca C, Martínez MJ, Cravero F, et al. Hybridizing Feature Selection and Feature Learning Approaches in QSAR Modeling for Drug Discovery /631/114/2248 /631/154/309 /639/638/563/606 /119/118 article. Sci Rep. 2017 Dec 1;7(1).
4. Ignacz G, Szekely G. Deep learning meets quantitative structure–activity relationship (QSAR) for leveraging structure-based prediction of solute rejection in organic solvent nanofiltration. J Memb Sci. 2022 Mar 15;646.
5. Tsou LK, Yeh SH, Ueng SH, Chang CP, Song JS, Wu MH, et al. Comparative study between deep learning and QSAR classifications for TNBC inhibitors and novel GPCR agonist discovery. Sci Rep. 2020 Dec 1;10(1).
6. Siegel RL, Miller KD, Wagle NS, Jemal A. Cancer statistics, 2023. CA Cancer J Clin. 2023 Jan;73(1):17–48.
